# Intraoperative Discovery and Laparoscopic Management of a Cholecystocolonic Fistula: A Case Report

**DOI:** 10.7759/cureus.103624

**Published:** 2026-02-14

**Authors:** Shoieb Mridha, Alice Hewitt, Niraj Khetan

**Affiliations:** 1 GI Surgery, Doncaster & Bassetlaw Teaching Hospitals NHS Trust, Doncaster, GBR; 2 General Surgery, Doncaster & Bassetlaw Teaching Hospitals NHS Trust, Doncaster, GBR

**Keywords:** bilioenteric fistula, cholecystocolonic fistula (ccf), chronic cholecystitis, gallstone disease, intraoperative management

## Abstract

Cholecystocolonic fistula (CCF) is a rare complication of chronic cholecystitis, characterised by an abnormal connection between the gallbladder and colon. It predominantly affects elderly patients with a history of gallstone disease and chronic inflammation. It is often difficult to diagnose preoperatively due to nonspecific symptoms, low sensitivity on current available imaging, and is often discovered intraoperatively. A high index of suspicion for a CCF should be maintained in patients with longstanding chronic cholecystitis, even when preoperative imaging is unrevealing. Surgeons should be prepared for altered anatomy and inflammation at operation and adjust their strategy accordingly to optimise patient outcomes. The laparoscopic approach is feasible and requires advanced laparoscopic expertise. Management varies from enterolithotomy in cases of emergency obstruction to fistula repair and cholecystectomy in elective cases. We report the case of a 63-year-old male with a history of gallstones and acute cholecystitis who underwent elective laparoscopic cholecystectomy. Intraoperatively, a CCF was identified and successfully managed via dissection and stapling of the fistulous tract with subsequent cholecystectomy. The patient recovered uneventfully and remained asymptomatic at follow-up.

## Introduction

Cholecystocolonic fistula (CCF) is an abnormal communication between the gallbladder and colon. It usually develops after longstanding inflammation, where recurrent cholecystitis leads to adhesions and gradual erosion into the adjacent bowel. It has been reported in 0.06% to 0.14% of patients with biliary disease [1,2]. Clinical presentation is often subtle, with intermittent abdominal pain, diarrhoea, or even minimal symptoms. This condition is mainly diagnosed intraoperatively during cholecystectomy, as preoperative imaging often fails to detect this rare entity because fistulous tracts are often small and radiologic findings can be nonspecific. Women are predominantly affected due to the higher prevalence of gallstones in this population, with most cases occurring in the sixth or seventh decade of life [3]. Failure to identify these fistulas during surgery can lead to severe complications, including inadvertent division of the fistula, unidentified colonic enterotomy resulting in faecal peritonitis, further surgery, stoma, and thus increased morbidity and mortality [3]. Our case report illustrates such a presentation. The importance of this case report lies in the demonstration of how a CCF can remain clinically silent and elude preoperative imaging. It emphasises the need for surgeons to anticipate such rare findings during cholecystectomy and adapt their operative strategy accordingly.

## Case presentation

We present the case of a 63-year-old man who was scheduled for an elective laparoscopic cholecystectomy due to gallstones. His past medical history included a small hiatus hernia, colonic diverticulosis, and fatty liver. He had no history of previous abdominal surgery. His anaesthetic risk assessment scale score was 2. He was on omeprazole for his dyspepsia symptoms. His BMI was 27 kg/m^2^.

**Figure 1:**
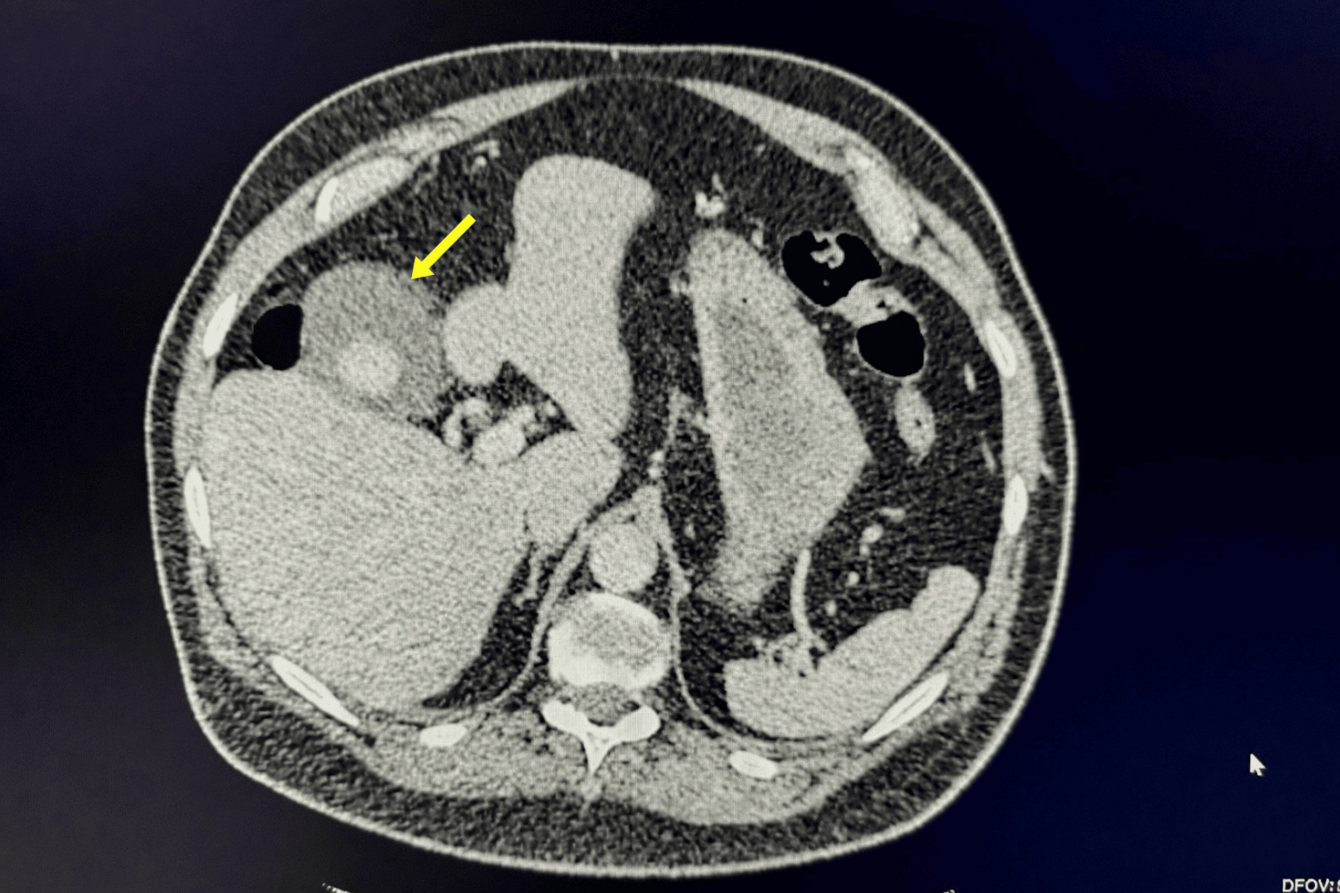
CT scan of the abdomen and pelvis showing inflamed gallbladder with gallstone

A year earlier, he presented to the emergency department with upper abdominal pain and elevated inflammatory markers. A CT scan of the abdomen and pelvis (Figure 1) revealed acute cholecystitis with gallstones but no pneumobilia or other features suggestive of a CCF. He was managed conservatively with intravenous antibiotics and analgesics and did not require further hospital admission. On subsequent clinic review, he reported only a few episodes of mild intermittent abdominal pain, with no other gastrointestinal symptoms such as diarrhoea, weight loss, or nausea. His physical examination was unremarkable. He was subsequently listed for elective laparoscopic cholecystectomy.

Patient’s preoperative laboratory investigations were within normal limits (Table 1), with no evidence of leukocytosis, cholestasis, or renal impairment. These findings were consistent with uncomplicated calculous cholecystitis and did not suggest biliary obstruction or active cholangitis.

**Table 1:** Pre-operative laboratory findings of the patient

Investigation	Patient value	Normal range	Interpretation
Haemoglobin	149 g/L	126-180 g/L	Normal
White cell count	10.9*10^9^/L	4-12*10^9^/L	Normal
Total bilirubin	10 umol/L	<21 umol/L	Normal
Alkaline phosphatase (ALP)	64 IU/L	30-130 IU/L	Normal
Serum creatinine	78 umol/L	64-104 umol/L	Normal

**Figure 2:**
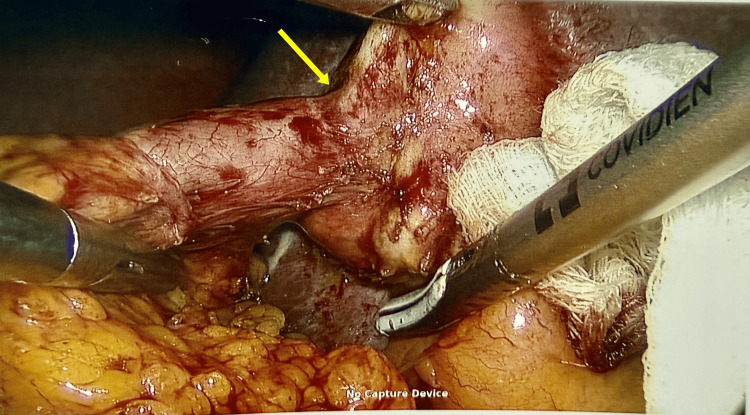
Cholecystocolonic fistula

A laparoscopic cholecystectomy procedure under general anaesthesia was perfomed with standard 4-port technique. The operative findings after careful dissection showed omental adhesions to the gall bladder, adhesions between the liver surface and the anterior abdominal wall. The gall bladder was contracted with distorted anatomy, especially with an unexpected finding of a cholecystocolic fistula involving the proximal transverse colon and body of the gall bladder (Figure 2).

**Figure 3:**
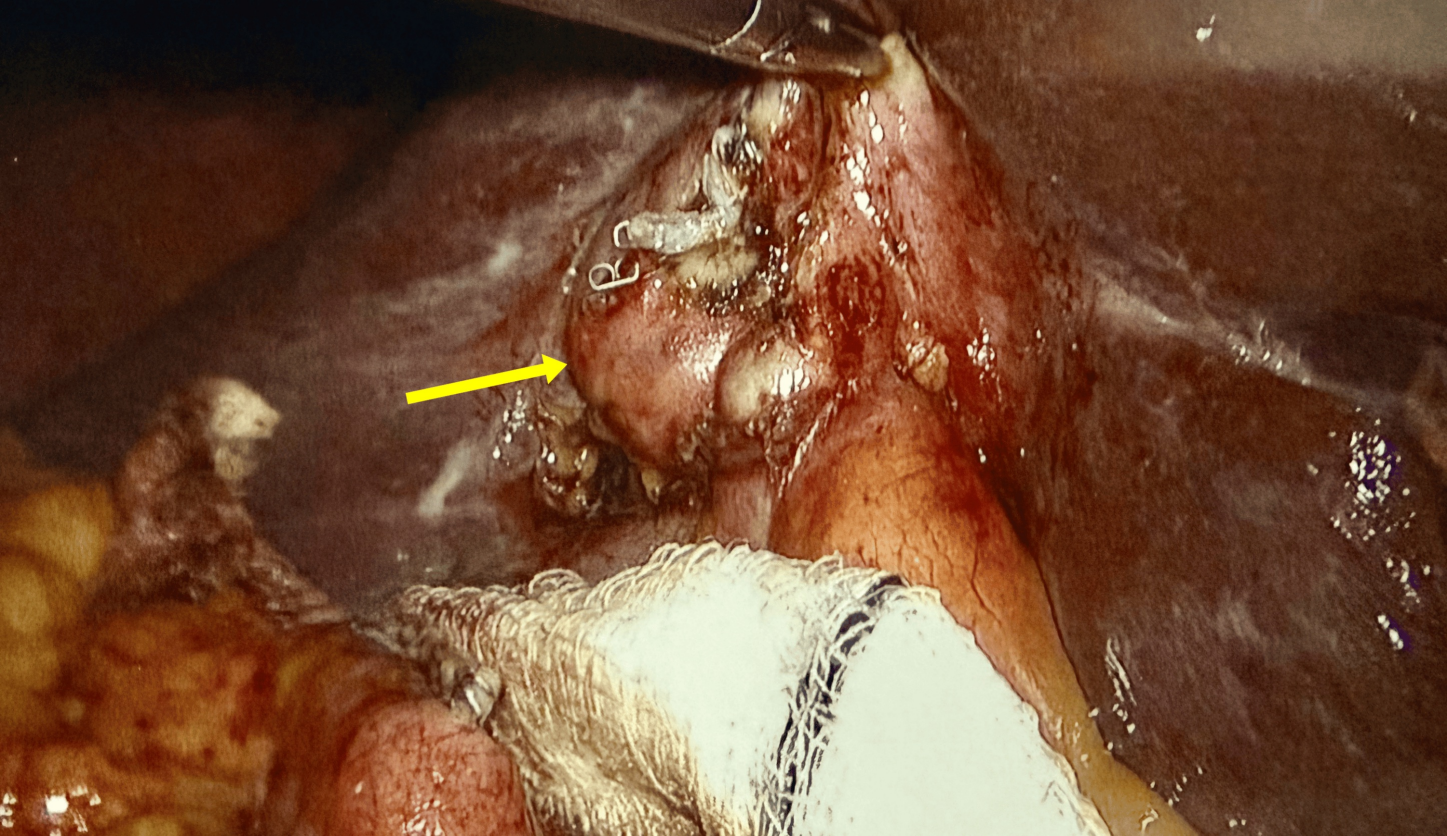
Contracted gallbladder with distorted anatomy

**Figure 4:**
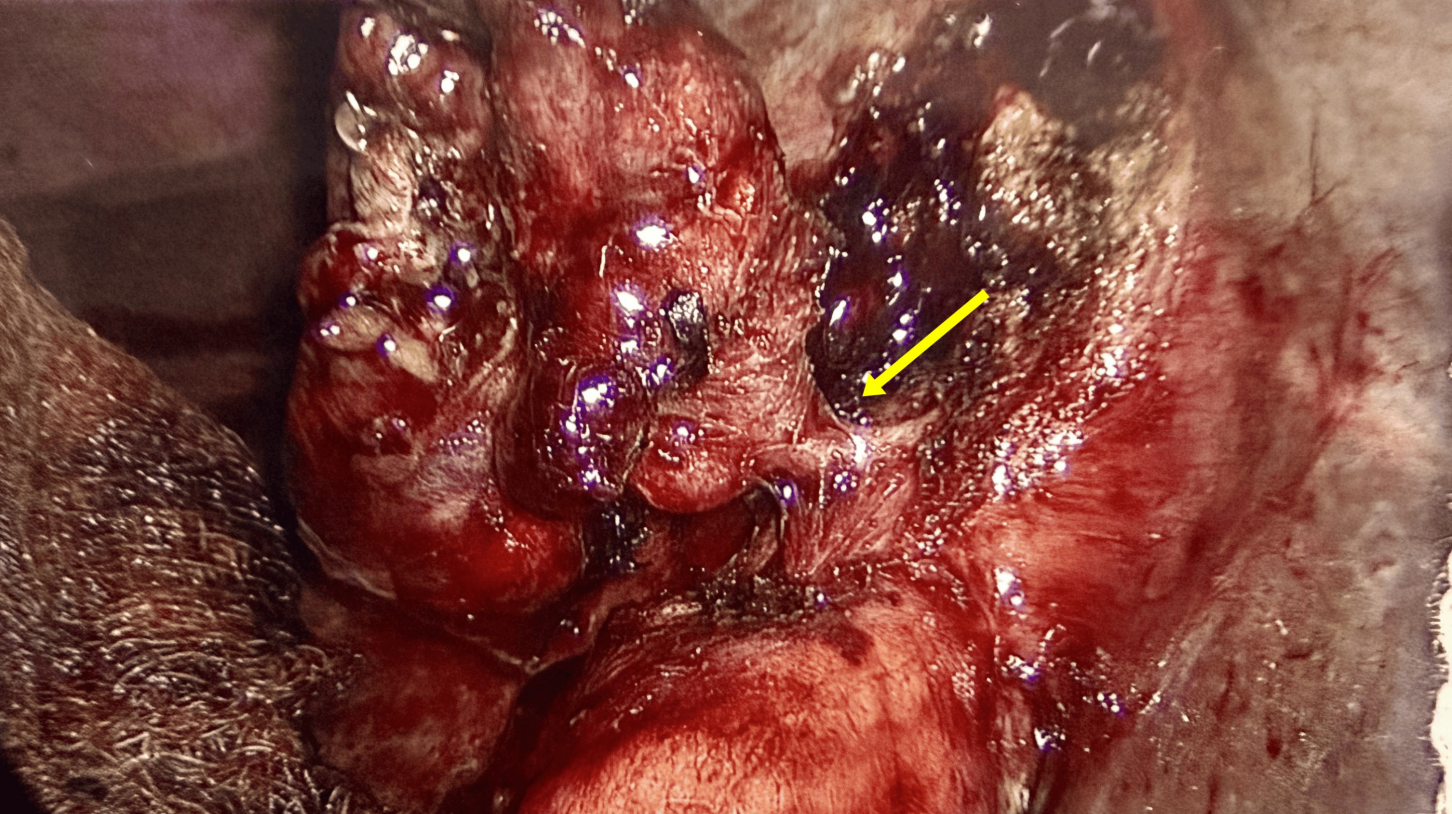
Cystic duct identified

**Figure 5:**
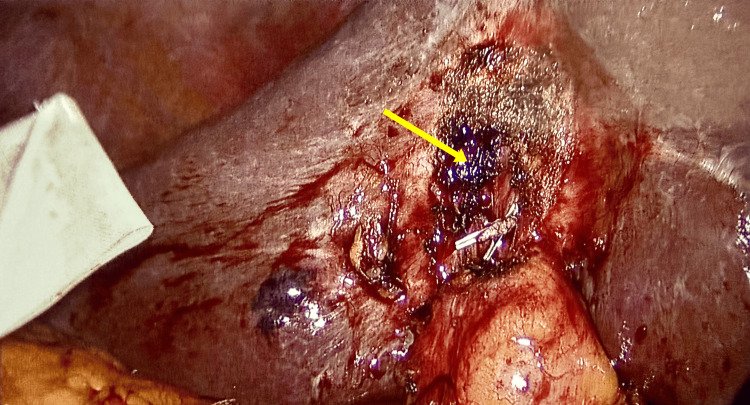
Liver bed after taking out the gallbladder

The surgery was facilitated by disconnecting the fistula at its apex on the colonic side using a linear laparoscopic stapler without compromising the colonic lumen. It further revealed a contracted gall bladder with distorted calot’s triangle (Figure 3). In view of that, gall bladder fundus was dissected first from liver bed. The branches of the cystic artery were carefully divided close to the gallbladder wall with ligasure, and duct was clearly identified, double clipped, and divided (Figures 4, 5). Haemostasis was achieved. A 20-French drain was placed in the right subhepatic space, and gallbladder was removed.

Postoperatively, the patient had an uneventful recovery. He was discharged on the second postoperative day with no complications and the drain was removed one week later. At four-week follow-up, he remained asymptomatic and was doing well.

## Discussion

A fistula is defined as an abnormal communication between two epithelial surfaces. When a fistula forms between the gallbladder and the bowel, it is referred to as a cholecystoenteric fistula. Two types of cholecystoenteric fistulas have been identified: cholecystoduodenal (the most common) and cholecystocolonic (the second most common) [3,4]. The cholecystoenteric fistula was first described by Courvoisier in 1890 [3]. CCFs occur more frequently in females, with a female-to-male ratio of approximately 2.47:1 [3]. Cases have been reported across a wide range of ages in both Western and Eastern populations; however, the condition is rarely observed in individuals under 50 years old [3].

Cholecystoenteric fistulas occur as a very rare and late complication of recurrent chronic cholecystitis. Chronic cholecystitis can cause severe complications, including empyema of the gallbladder, gallbladder perforation, cholecystoenteric fistula formation, or, as illustrated here, CCF. CCFs can also arise in patients with inflammatory bowel disease or malignancies affecting the biliary system, colon, or pancreatic head [3,5].

The development of CCF as a complication of gallstone disease is not fully understood. Two main hypotheses have been proposed. One theory suggests that during an episode of acute calculous cholecystitis, inflammation causes the gallbladder to adhere to the adjacent colon. As the inflammation progresses, ischemia may lead to gallbladder wall necrosis. Increased internal pressure within the gallbladder then causes its contents to erode through the necrotic tissue and into the adjacent colonic wall, eventually creating a fistulous tract. An alternative hypothesis is that a gallstone becomes lodged, typically in the gallbladder neck, resulting in sustained pressure that causes localised tissue breakdown and subsequent fistula formation between the gallbladder and colon [3,6,7].

CCF may present with vague or nonspecific symptoms such as abdominal discomfort, nausea, unintended weight loss, diarrhoea, and signs of malabsorption. A proposed diagnostic triad of pneumobilia, chronic diarrhoea, and vitamin K deficiency is potentially pathognomonic for CCF [8]. In patients with calculous chronic cholecystitis and an additional history of ascending cholangitis, gallstone ileus, or obstructive jaundice, clinicians should maintain a high index of suspicion for a possible fistula. In our case report, the patient presented with calculus cholecystitis and intermittent minimal pain before surgery, with an interval of 12 months between presentation and surgery. Delayed surgery in patients with cholecystitis can be a contributing factor for fistula formation [3]. 

CCF is mainly diagnosed intraoperatively due to its rarity. There is no single definitive investigation to diagnose it preoperatively. In selected cases, abdominal X-rays, ultrasound, barium studies, biliary scintigraphy, and endoscopic retrograde cholangiopancreatography (ERCP) can aid diagnosis [3,8,9]. Although pneumobilia on plain abdominal radiographs is traditionally considered a key indicator of bilioenteric fistula, this finding is not consistently present [3]. In cases where a fistula is suspected during surgery, intraoperative cholangiography can be used to confirm the diagnosis [3]. Among preoperative imaging modalities, ERCP is regarded by some researchers as the most reliable tool for detecting CCF [8]. 

In our case, preoperative imaging demonstrated only acute calculous cholecystitis without evidence of fistula formation, and the patient reported minimal intermittent pain. Despite normal laboratory values and subtle symptoms, the CCF was only identified intraoperatively, highlighting the tendency of CCF to remain clinically silent and radiologically occult. The failure of preoperative imaging to detect the fistula in this case may be attributed to several factors. Small or intermittently patent fistulous tracts may not generate sufficient pneumobilia or contrast passage to be visualised on routine imaging modalities. Additionally, chronic inflammation and dense adhesions between the gallbladder and adjacent colon can obscure anatomical planes, making differentiation between inflammatory changes and true fistulous communication difficult on ultrasound or CT [3,4,8,9]. The prolonged interval of 12 months before definitive management may have allowed ongoing chronic inflammation, contributing to fistula formation.

CCF can lead to large bowel obstruction. In most cases, a gallstone becomes impacted usually in the rectosigmoid colon, often within a segment of diverticular disease, especially if the stone exceeds 2.5 cm in diameter [10]. Smaller stones typically pass through the colon and are excreted without any obstructive symptoms [11]. Obstructions usually occur in the presence of pre-existing bowel narrowing, with diverticular disease being the most frequently associated condition [12].

The management of CCFs and their sequelae remains controversial, with no clear consensus. Patients who are asymptomatic and frail can be treated conservatively [3]. In cases where colonic obstruction is present, the preferred approach is enterolithotomy [3]. Additionally, it is essential to explore both the small and large bowel intraoperatively for additional stones to prevent re-obstruction [7].

The decision to repair the bilioenteric fistula and perform cholecystectomy during the same procedure remains debated. Some surgeons advocate for a one-stage approach, performing both cholecystectomy and fistula repair during the initial surgery, while others prefer a two-stage approach, deferring cholecystectomy until after the patient has stabilised [3,9]. In some cases, cholecystectomy may even be omitted altogether [6].

Laparoscopic treatment is a viable option for elective cases when performed by experienced surgeons; however, it may be associated with longer operating times and higher open conversion rates [4]. Advanced skills such as laparoscopic suturing, advanced energy sources such as Ligasure, and laparoscopic stapling devices facilitate and improve success with minimal invasive surgery, as demonstrated by our case. In selected cases, the use of indocyanine green fluorescence cholangiography can facilitate safe surgery [13]. Compared with open surgery, laparoscopic management has been associated with reduced postoperative pain, shorter hospital stay, earlier mobilisation, and faster recovery, provided that adequate expertise and intraoperative judgment are exercised.

Robotic cholecystectomy has emerged as an alternative to conventional laparoscopy for challenging biliary surgery. The robotic system’s high‑definition 3D visualisation and articulated instruments provide enhanced dexterity and precision during dissection, which may facilitate safer surgery in cases with distorted anatomy or complex inflammation [14].

## Conclusions

CCF is uncommon due to nonspecific clinical presentation and challenges in preoperative detection; however, surgeons should maintain a high index of suspicion, particularly in patients with longstanding gallbladder pathology. As demonstrated in this case, preoperative recognition may be minimal, and diagnosis is often made intraoperatively. Surgical management should be individualised based on intraoperative findings, patient stability, and surgeon experience. When feasible, laparoscopic repair offers a minimally invasive option but requires meticulous dissection due to inflammatory changes and distorted anatomy. Early diagnosis and timely surgical intervention are key to reducing morbidity and ensuring favourable patient outcomes. Also, the use of advanced laparoscopic techniques and instruments facilitates surgery.
